# Global Tanning Bed Advertising: A Comparison of Legal Regulations on Three Continents

**DOI:** 10.3390/cancers15174362

**Published:** 2023-09-01

**Authors:** Sonja Mathes, Karla S. Lindwedel, Lill Tove Nilsen, Isabelle Kaiser, Annette B. Pfahlberg, Olaf Gefeller

**Affiliations:** 1 Department of Dermatology and Allergy, Technical University of Munich, 80802 Munich, Germany; sonja.mathes@mri.tum.de; 2 Department of Medical Informatics, Biometry and Epidemiology, Friedrich-Alexander-Universität Erlangen-Nürnberg, 91054 Erlangen, Germany; karla.lindwedel@fau.de (K.S.L.); isabelle.kaiser@fau.de (I.K.); annette.pfahlberg@fau.de (A.B.P.); 3 Section for Environmental Monitoring and Radon- and UV-Protection, Norwegian Radiation and Nuclears Safety Authority, NO-1332 Østeras, Norway; lill.tove.nilsen@dsa.no

**Keywords:** advertising, legislation, sunbathing, sunbed, tanning bed, tobacco control, ultraviolet radiation

## Abstract

**Simple Summary:**

Epidemiologic studies showed that ultraviolet radiation from tanning beds causes skin cancer. Many countries now have laws regulating commercial indoor tanning, including rules on advertising and information for customers. We compiled a comprehensive overview of these regulations in 131 legislative units across North America, Australia/New Zealand, and Europe. The type and content of legal restrictions varies widely. In 49 legislative units, we identified different types of advertising bans for indoor tanning, while 64 legislative units had regulations mandating the dissemination of specific health information to tanning bed customers. In nearly 40% of the legislative units, there was no legislation at all on these topics. The heterogenous results call for an international dialogue between health authorities and governments to harmonize the regulatory framework for tanning bed advertising and information requirements to a level better protecting the public from skin cancer.

**Abstract:**

Artificial ultraviolet radiation from tanning beds has been classified as carcinogenic by the International Agency for Research on Cancer in 2009. Several countries have subsequently introduced comprehensive legislation regulating commercial indoor tanning. Specific aspects of these regulations address tanning bed advertising and information requirements for tanning bed customers, which have been previously neglected in international comparisons of indoor tanning regulations. We performed a systematic search regarding legislation on these aspects in 131 legislative units across three continents (North America, Australia/New Zealand, Europe). The legal restrictions found varied widely in type and content. In 49 legislative units we identified total (*n* = 8) or partial legal bans (*n* = 41) on advertising for indoor tanning, while 64 legislative units enacted 5regulations that necessitate the dissemination of different types of specific health information to tanning bed customers. Nearly 40% of the legislative units of the study region lacked any legislation on these issues altogether. The heterogenous results emphasize the need for an international dialogue between health authorities and governments to harmonize the regulatory framework for tanning bed advertising and information requirements to a level better protecting the public from skin cancer. Our comprehensive international comparison can serve as a starting point for such a harmonization process that may ultimately protect the public worldwide from misleading tanning bed advertising.

## 1. Introduction

Ultraviolet radiation (UVR)-associated skin cancers, mainly malignant melanoma and keratinocyte carcinoma, are of increasing concern to healthcare systems worldwide [1]. Melanoma incidence showed a steady annual increase of around 3–7% over the past decades in predominantly fair-skinned populations, and it is expected that this trend will continue [2]. The incidence of keratinocyte carcinoma, i.e., squamous cell carcinoma and basal cell carcinoma, has also been steadily increasing since the middle of the last century, and projections from age–period–cohort modeling suggest a further sharp increase in incidence over the next decade [3].

The main driver of this development was the change in UVR exposure on the population level [4]. Solar and artificial UVR exposure from tanning beds, the latter classified as carcinogenic by the International Agency for Research on Cancer (IARC) in 2009 [5], have been found to be involved in the development of malignant melanoma of the skin and keratinocyte carcinoma [6,7]. Even more pronounced this has also been confirmed for early-onset skin cancer that has more often dramatical consequences for the individuals concerned [8]. Strong intermittent UVR exposure as well as high cumulative UVR exposure have long been established as relevant risk factors relevant for the development of skin cancer [9]. Data support the idea that these patterns of UVR exposure increase skin cancer risk, particularly when exposed at a young age [10,11,12,13,14,15].

Reducing UVR exposure from tanning beds could, therefore, contribute to a decrease in the incidence of skin cancer. Tanning bed use is relevant for the total population, but younger people are the primary focus of the tanning bed industry and successfully targeted through advertisements promoting tanning bed use [16,17]. In the Euromelanoma study conducted in 30 European countries, involving 227,888 participants, 6% of adolescents (≤19 years old) and 17% of young adults (20–35 years old) reported use of indoor tanning, while this behavior was less common in the elderly [18]. Similar prevalence studies in North America revealed an even higher usage of indoor tanning among adolescents [19].

As with health issues in general, there is an opportunity for official government bodies to intervene in the prevention of skin cancer through regulatory measures. This can be achieved directly through legislation imposing bans on indoor tanning. Total and partial bans for minors have proven to be effective in reducing artificial UVR exposure, especially among female adolescents [20,21,22].

Regulations for the tanning bed industry vary internationally. Brazil and Iran were the first countries to introduce a total ban of tanning devices in 2008/2009 [23,24]; only Australia, which has both a high environmental UVR exposure and a predominantly fair-skinned population, followed in 2015/2016 [25,26]. Unlike Australia, many other countries with a fair-skinned population do not have strict access restrictions to tanning salons, not even for minors [27,28,29].

In recent years, there has been a global political trend away from bans and other strict legal regulation, enforced by assertive state authorities, towards softer instruments to guide and manage health behaviors [30]. This includes more decentralized control through “governance” that operates through indirect influence rather than prohibitions. Such more lenient regulations comprise voluntary industry agreements and introduction of product labeling, as well as market instruments, such as taxation and various advertising regulations [30]. Advertising bans have been utilized successfully in the public health context of tobacco control to positively influence health-related consumer behavior [31].

A systematic overview of international regulations on advertising for commercial indoor tanning and information requirements addressing tanning bed users is currently lacking. This study provides the first compilation of international legislation on such regulations for the tanning bed industry on three continents, North America (the US and Canada), Australia/New Zealand, and Europe.

## 2. Materials and Methods

### 2.1. General Search Procedure

We employed a comprehensive web search strategy to collect data on the official legislative demands for the operation of tanning salons in North America (the US and Canada), Australia/New Zealand, and Europe. This study focuses on the regulations governing the advertising of tanning salons and the requirements for the disclosure of information to their customers. We used the search function in Google’s Chrome browser allowing for direct translation of search terms and results into different languages. Our inquiry began with the phrases “tanning bed” and “legislation”, translated into the appropriate national language alongside the relevant country name. If the first results page failed to include an official government website, we added “site:.gov.country code” as an additional filter in the search bar. If this was not successful either, relevant news magazine entries among the initial search results were scanned for official legislative sources and terms for regulations. A targeted search was then conducted in the official documents that were found via this search. If this was not sufficient to identify suitable regulations, more intensive individual Google searches were conducted for the countries in question. All text material found was translated using Google translate to facilitate the evaluation for the predominantly German research group and to increase comparability between texts. After translation, all text passages identified as relevant were transferred to an Excel spreadsheet (Microsoft Office Professional Plus 2019). The search activities were conducted between March 2021 and January 2022. All results found during the search activities were then cross-checked with information from the WHO Global Health Observatory data repository on national regulations concerning public information and safety of sunbeds [32]. This WHO information resource, last updated in 2017, covers some of the aspects addressed here in less detail. In case of discrepancies between the information in the WHO data repository and our own findings, we reevaluated the relevant legislative document and used the most recent information available. From February to June 2023, we updated our search results regarding tanning bed advertising and information requirements to ensure an up-to-date compilation of legal regulations on these topics.

### 2.2. Exceptions to the General Search Procedure

For the US and Canada, there were particularities in seeking regulations of tanning bed advertising. In the case of the US, we obtained information mainly from the website of the AIM at Melanoma Foundation (https://www.aimatmelanoma.org/legislation-policy-advocacy/indoor-tanning/, accessed on 29 August 2023). Most relevant sources for legislation on tanning beds in US states could be found via this site, and the rest directly through Google searches. For Canada, the recent work by Gosselin and McWhirter [33] replaced keyword searches as the primary source of information for identifying legal requirements for tanning bed advertising. The study provides an overview of the relevant legislative requirements in Canada with the corresponding laws accurately identified.

### 2.3. Presentation and Findings

We present the results on legal regulations of tanning bed advertising and information requirements as follows: First, we classified bans of tanning bed advertising as explicit total, implicit total, and partial. Implicit total bans exist in regions where commercial tanning beds are prohibited entirely. Explicit total bans on advertising prohibit advertisements of all tanning salons in regions that do not completely ban commercial indoor tanning. Partial bans prohibit specific forms of advertising, are targeted to specific audiences, or exclude specific topics in the advertisement such as health-related claims. Health-related claims are statements that imply positive health effects of tanning bed usage, for instance by touting a rise in the serum levels of vitamin D. They are distinguishable from other types of advertising content as they do not include statements that refer to attractiveness only. We compiled a list of regions imposing explicit and implicit total as well as different types of partial bans. Additionally, we reviewed legislative obligations in regions without a total ban to disclose information about the health risks associated with indoor tanning, which is considered a less restrictive instrument of regulation and can be required as part of advertising and/or as mandatory on-site information in tanning salons. These obligations require the dissemination of specific health information that emphasizes the vulnerability of special groups, such as fair-skinned individuals who use tanning beds or other users with unique characteristics that could increase the harm from indoor tanning. We provide a comprehensive overview of where such health information must be disseminated by legal requirement and how this must be carried out. Appendix A contains all relevant source data required for analysis (see Table A1).

## 3. Results

We found a heterogeneous picture of legislative regulations on tanning bed advertising and information requirements in the study region which encompassed a total of 131 legislative units. In 49 of those units, there are either total (*n* = 8), explicit total in one case and implicit total in seven cases, or partial bans (*n* = 41) on advertising for tanning salons. Legislative units, referring to geographical areas with their own independent legislation (primarily countries in Europe, states in the US, states and territories in Canada and Australia/New Zealand), enacting bans or other legislation were found in all parts of the study region. As a less restrictive alternative to bans, 64 legislative units enacted regulations to require dissemination of specific health information to tanning bed users, rarely as a mandatory component of advertising, more often as part of on-site information for tanning salon customers. In 32 legislative units, we identified partial bans and other additional regulations implemented concurrently.

### 3.1. Bans on Advertising

In one country, Poland, we found an explicit total ban on advertising for artificial tanning devices. As all Australian states and territories have completely banned commercial indoor tanning, there is no specific legislation on tanning bed advertising. Therefore, we classified all seven Australian states and territories in Table 1 as having enacted an “implicit total ban”. All other legislative units have either no or only partial bans on advertising for tanning salons.

Partial bans refer to specific target groups or types of content in advertising. We found partial bans on advertising in 41 legislative units across all three continents (see Table 1). The majority of regions with partial bans are located in the US (*n* = 22). The two most common restrictions on indoor tanning advertising were the requirement to avoid overall health-related (*n* = 32) and “free from risk” claims (*n* = 22) associated with indoor tanning. Often, these two types of partial bans were coupled (*n* = 19). Seven Canadian legislative units did not allow advertising specifically directed at minors, while seven US states forbid reference to certificates that confirm compliance with the technical specifications of the Food and Drug Administration (FDA) for tanning beds in advertisements for indoor tanning. One European country, Ireland, banned special offers and bonus promotions designed to encourage repeated visits to tanning salons.

### 3.2. Information Requirements

In five European countries (France, Latvia, Luxembourg, Portugal, and Spain), all tanning bed advertising must include a prescribed health warning. Legislation has also introduced mandatory information requirements on the health risks associated with tanning beds beyond advertising. The use of this regulatory instrument requires that the relevant health information must be disseminated in a legally prescribed manner on site at the tanning salon. In 19 legislative units (13 in Europe, 6 in the US), displaying the information on-site is legally sufficient. In 43 legislative units (13 in Europe, 30 in the US), the requirement of an on-site display is coupled with the necessity of providing written handouts disseminating the health information to all tanning bed customers. More than two thirds of these legislative units (*n* = 29) require a signed acknowledgment of receipt from the customer. In two further European countries, written handouts that require the customer’s signature as confirmation are the only mandatory means of disseminating health information, an on-site display is not required. In addition to these written forms of conveying health information to the customers, 10 legislative units (9 in Europe, 1 in the US) require additional oral information.

Table 2 displays the details of all 64 legislative units that have specific own legislation on this aspect. The contents of the mandatory information about health risks of tanning bed use varies between regions. For the US and Europe, superordinate federal and international organizations provide additional guidance on information disclosure about health risks linked to tanning bed usage. The FDA regulation 21CFR1040.20 [34] mandates that tanning salons in all US states must display a warning statement utilizing a prescribed text. The statement pertains to possible eye and skin injuries resulting from tanning bed use and identifies premature skin aging and skin cancer as hazards of repeated exposure. The health warning includes the risk for individuals using medications and cosmetics that may increase sensitivity to UV radiation. The specific risk for individuals with a history of “skin problems” and “sensitive to sunlight” is explicitly indicated. Eight US states have enacted legislation on tanning bed use that comprises information requirements addressing specific subgroups in even more detail. The subgroups of pregnant women, those with melanoma cases in the family history, and those with a large number of nevi have to be addressed by health warnings according to these regulations.

For most European countries, the European Electrotechnical Committee for Standardization (CENELEC) has issued the standard EN 60335-2-27 covering tanning bed use to which all 33 CENELEC members adhere [35]. EN 60335-2-27 specifies detailed information requirements and health warnings for specific vulnerable subgroups. Individuals “under the age of 18”, “with a tendency to freckle”, “with natural red hair color”, “with abnormal discolored patches on the skin”, “with a large number of moles”, “with asymmetrical irregularly shaped moles larger than 5 mm in diameter with variable pigmentation and irregular border”, “suffering from sunburn, who are unable to tan or burn easily”, “who have a history of frequent and severe sunburn during childhood”, “suffering or previously suffering from skin cancer or who are predisposed to skin cancer”, “under a doctor’s care for diseases that involve photosensitivity” and “reactive to photosensitizing medicine” are advised against tanning bed use. In 21 European countries, information requirements addressing tanning bed customers are included in specific legislation in these countries. In many of these cases the EN 60335-2-27 specifications are restated, sometimes in abbreviated form. However, nine countries have appended the subgroup of pregnant women and two the subgroup of breastfeeding women to the inventory of tanning bed customers who should specifically be informed about the hazards of tanning bed use. Neither in the Canadian states and territories nor in New Zealand’s legislative units did we find any regulation on the mandatory dissemination of health information to tanning bed customers.

## 4. Discussion

We found some type of legal regulation of tanning bed advertising and/or information requirements for disseminating health information to tanning salon customers in 81 legislative units, representing over 60% of the total 131 legislative units in our study region, which included North America, Australia/New Zealand, and Europe. The legal regulations varied in their strictness and were highly diverse. Notably, Poland is the only legislative unit that has enacted a total ban on all such advertising, though commercial indoor tanning remains permissible in the country (except for minors). Half of the legislative units enacting regulations had enforced partial bans on tanning bed advertising. Due to a large number of US states having these regulations, predominantly health-related and “free from risk” claims have legally been banned in advertising. The remaining legislative units had some form of a more lenient legal regulation that only required disseminating health information for specific vulnerable groups in advertisements and/or on-site at the tanning salons.

More than a decade ago, the IARC, an intergovernmental agency that is part of the World Health Organization (WHO), classified artificial UVR as a group I carcinogen, indicating that there is strong evidence for its carcinogenicity in humans [5]. The WHO also recommended that governments enforce legally binding regulations for the operation of tanning beds [36,37]. One aspect of this regulation concerns tanning bed advertising. To date, only three countries (Brazil, Iran, Australia) have completely banned commercial indoor tanning, thereby eliminating any basis for advertising. Several international comparisons have already been conducted on legal regulations for commercial indoor tanning and customers’ compliance with these regulations [27,28,29,38,39,40,41]. However, none of these studies have examined tanning bed advertising and information requirements which have been the focus of our investigation. In countries that do not completely ban indoor tanning, there are options to impose either stricter or more lenient legal regulations, with advertising specifications being just one element [30]. Possible government interventions beyond advertising restrictions comprise calls for voluntary industry commitments of good conduct, the introduction of taxation as well as the implementation of mandatory certification systems and labeling requirements for UVR protection in tanning salons. On-site confrontation of customers with facts about skin cancer as well as broad educational campaigns can serve as further means of governance to influence behavior [30].

The idea that governmental restrictions of advertising for carcinogenic substances can benefit public health is exemplified by the case of cigarette smoking. Scientific evidence established that tobacco is an addictive and harmful substance long before the implementation of global legal restrictions on the advertising of tobacco products [31]. Germany, for example, adopted regulations on tobacco advertising in several stages. In 1975, a ban on television and radio advertising for tobacco products was implemented. Restrictions on cinema commercials followed in 2002, and from 2007 newspaper and internet advertising was abandoned. In parallel, smoking prevalence has declined over the last decades in Germany, and this trend is ongoing [42].

Indoor tanning shares similarities with the health issue of smoking in other aspects, including the targeting of a young audience for advertising. The tanning bed industry uses widespread advertising on social media focusing on adolescents and young adults [16,17]. As the skin of children and adolescents is particularly susceptible to UVR damage, advertising for tanning beds targeted at this age group needs to be subject to critical evaluation. Minors have shown particular interest in using tanning beds, when accessible for them [43]. We found legal restrictions on tanning bed advertising specifically targeting minors only in seven Canadian states and territories, suggesting that there is still significant progress to be made in safeguarding minors.

Interesting parallels also emerge from addictive effects known for tobacco consumption as well as of tanning bed use. UV light has been shown to be addictive, with approximately 10% of tanning teens showing signs of addiction [44]. The mechanisms of addictive effects of UVR have been described [44]. Central endorphin release, central nervous regulation of opioid receptors, and local proopiomelanocortin-mediated beta-endorphin release in the skin itself play a role in the creation of the addictive effect of UVR [45,46,47]. While the addictive effects of smoking have played some role in discussions of legislative approaches to tobacco control in the past, little attention has been paid to this issue regarding the regulation of tanning beds.

Even though there are many parallels between tobacco use and indoor tanning, the current international situation of advertising restrictions is dramatically different. While direct tobacco advertising has long been banned in most parts of the world [48], legal regulations for tanning bed advertising are far more lenient. The tanning bed industry employs a similar tactic to advertise its health-hazardous products and evade more stringent regulations as the tobacco industry did a few decades ago [49,50].

Forbidding health-related claims has been identified in our study as the most prevalent partial ban on tanning bed advertising. Positive health effects of indoor tanning, specifically the production of vitamin D induced by indoor tanning, is used by the tanning bed industry for advertising in regions that do not prohibit health-related claims. Endogenous production of vitamin D in humans depends on UVR exposure of the skin [51]. In most regions of the world, a sufficient vitamin D supply can be attained by regularly exposing roughly 10% of the body surface, for example represented by both forearms, to solar UVR for a period of 5 to 20 min, depending on skin type and season [52,53,54]. Even when under extreme conditions, vitamin D supply cannot fully be maintained using solar UVR, benefits from using tanning beds with respect to vitamin D production do not outweigh the cancer risk associated with indoor tanning [53,55].

The use of artificial UVR has undeniable health-promoting effects when medically applied. For example, UVR phototherapy is successfully used to treat numerous inflammatory skin diseases. In medical applications, the indication for exposure to the different wavelengths (UVA1, UVB narrow band) depends on the underlying disease and the individual patient’s medical history. Here, in contrast to the less standardized use of commercial tanning beds, schemes that have been tried and tested over many years and are constantly being improved are utilized with an optimized duration of UVR exposure [56]. In addition, narrow, defined spectra of UVR are used, with the idea of maximizing the healing effect while keeping the risk low. In medical applications, physicians choose the UVR dosage below the patient’s minimal erythemal dose (MED) and strictly consider contraindications and management of side effects [57]. In contrast, commercial tanning devices can emit irradiation intensities equivalent to a UV index of 36, causing one MED to be surpassed in very short time [58].

In the majority of the legislative units surveyed, tanning salon operators must comply with mandatory information disclosures. These disclosures inform customers of health risks associated with tanning beds, especially vulnerable subgroups at an increased risk of skin cancer. The prescribed content and the dissemination method of this health information varies from region to region. Even more problematic than this heterogeneity is the practical implementation of these legal requirements. A recent study from the US found that customers in a nationally representative sample of tanning salons still receive potentially dangerous and convoluted information about tanning bed safety from salon operators [59].

Our investigation has limitations that should be kept in mind when using our results. We cannot guarantee that we have not overlooked legislative regulations on tanning bed advertising, despite our extensive search for relevant web sources in all legislative units of the study region. It is possible that some countries do not publish their legal regulations online or that locating the corresponding website is hindered by language barriers. In 14 European countries, we were unable to locate any current legislation on tanning beds, indicating the absence of restrictions on tanning bed advertising in those countries. Intensive efforts to verify the absence of specific tanning bed legislation were successful for seven countries. In these cases, independent verification by health authorities and/or embassies of the corresponding countries or by local media articles on the tanning bed industry was available. Another complicating issue is the differing responsibility for legislation on tanning bed advertising among various ministries in the legislative units of the study regions. For example, a ministry of consumer protection may enact laws on general legal aspects of advertising that also contain specific provisions regulating tanning bed advertisements. These provisions may not be immediately apparent from the titles of the laws. Such legislative regulation would not have been found without explicit media coverage of the issue in the corresponding country. By design, our investigation did not cover the entire world, we searched only 131 legislative units on three continents. We excluded Asia, Africa, and South America, because we expected to find only a handful of countries with legislation on tanning bed advertising, and we felt that verifying the absence of such laws in these regions would require extensive effort with limited information gain.

Legislation is always a dynamic system that will change over time. Our compilation describes the current state of legislation and needs continuous updating. To this end, a living data repository, ideally under the auspices of an international organization such as the WHO, providing this information in an always up-to-date version would assist policy makers in keeping track of the current state of tanning bed legislation and information requirements around the world. The existing WHO Global Health Observatory data repository on national regulations for tanning beds [32] should be extended and continuously updated to fulfill this task.

## 5. Conclusions

Our comparison of the legal framework for tanning bed advertising and information requirements on three continents revealed a heterogeneous picture of regulations that are far less restrictive for the tanning bed industry than for the tobacco industry with regard to smoking advertising. The findings indicate a need for health authorities and governments to engage in an international discussion in order to harmonize the regulatory framework for tanning bed advertising and information requirements to better protect the public. Our comprehensive compilation of international regulations may serve as a starting point for such a harmonization process that may ultimately stop worldwide misleading tanning bed advertising and incomplete information of customers of tanning salons. This effort will significantly contribute to a reduction in the risk of UVR-associated skin damage.

## Figures and Tables

**Table 1:** Overview of legislative units in the study region with bans (explicit total, implicit total, partial) on tanning bed advertising. Legislative units are grouped into four subregions (Europe, US, Canada, Australia/New Zealand). Absolute and relative frequencies for the different types of bans are given.

Advertising Bans (All)	*n* (%)	Europe	US	Canada	Australia/New Zealand
Number of legislative units with any type of ban	49 (100)	12	22	7	8
Total ban (explicit)	1 (2)	Poland	-	-	-
Total ban (implicit)	7 (15)	-	-	-	New South Wales Northern Territory Queensland South Australia Tasmania Victoria Western Australia
Partial ban (all)	41 (84)	11	22	7	1
Health related claims	32 (65)	France Ireland Latvia Lithuania Luxembourg Northern Ireland Portugal Romania Slovenia Spain Sweden	California Florida Georgia Idaho Illinois Iowa Kansas Louisiana Michigan New Jersey North Carolina North Dakota Oklahoma Oregon Texas Virginia Washington Wisconsin	New Brunswick Quebec	Auckland
“Free from risk” claims	22 (45)	Spain	California Florida Georgia Idaho Illinois Indiana Iowa Kansas Louisiana Massachusetts Michigan New Jersey North Carolina North Dakota Oklahoma Oregon Texas Virginia Washington Wisconsin	-	Auckland
Advertising to minors	7 (14)			Alberta Manitoba New Brunswick Ontario Prince Edward Island Saskatchewan Quebec	-
Use of certificates	7 (14)	-	Maine Mississippi New Jersey North Carolina North Dakota Oregon Wisconsin	-	-
Bonus programs	1 (2)	Ireland		-	-

**Table 2:** Overview of those 64 legislative units in the study region regulating the type of dissemination of written and oral health warnings for tanning bed customers. Legislative units are grouped into only two subregions (Europe, US) as no legislative units from the other two subregions (Canada, Australia/New Zealand) use this instrument. Absolute and relative frequencies for the different subgroups are given.

Health Warnings Disseminated to Customers by	*n* (%)	Europe	US
On-site displays in tanning salons and obligatory handouts for customers (with signed acknowledgment of receipt)	29 (45)	Belgium Latvia Lithuania Portugal Romania Serbia Spain	Arizona Florida Illinois Indiana Iowa Massachhusetts Michigan Nevada New Hampshire New York North Carolina North Dakota Ohio Oklahoma Oregon Pennsylvania Tennessee Texas Virginia Washington West Virginia Wisconsin
On-site display in tanning salons and obligatory handouts for customers (without signed acknowledgment of receipt)	14 (22)	Austria France Germany Luxembourg Scotland Wales	California Colorado Delaware Hawaii Kansas Kentucky Louisiana Maine
On-site displays in tanning salons only	19 (30)	Denmark England Finland Iceland Italy Monaco Netherlands Norway Poland Slovakia Slovenia Sweden Switzerland	Georgia Maryland Minnesota Mississippi Nebraska New Jersey
Obligatory handouts for customers (with signed acknowledgment of receipt)	2 (3)	Ireland Moldova	
Obligatory oral instructions additional to on-site display and handout	10 (16)	Belgium Italy Latvia Moldova Netherlands Portugal Romania Spain Switzerland	New Hampshire
Total	64 (100%)	28	36

## Data Availability

The data are included in the article.

## Appendix A

**Table A1:** List of all 81 legislative units with regulations for advertising for commercial indoor tanning and/or information requirements. In addition to categorizing the type of legal regulation, the table contains the original names of relevant legislation. The remaining 50 legislative units surveyed in which neither bans nor specific regulations for the dissemination of health information could be found were (in brackets, the indication to which study region the legislative unit belongs: AUS: Australia, CN = Canada, EU = Europe, NZ = New Zealand, US = United States, * indicates that this country is CENELEC member and thus EN 60335-2-27 applies; FDA regulation 21CFR1040.20 applies to all U.S. states): Alabama (US), Alaska (US), Albania (EU), Arkansas (US), Andorra (EU), Bay of Plenty (NZ), Belarus (EU), Bosnia (EU), British Colombia (CA), Bulgaria* (EU), Chatham Islands (NZ), Connecticut (US), Croatia* (EU), Cyprus* (EU), Czech Republic* (EU), Estonia* (EU), Greece* (EU), Gisborne (NZ), Hawke’s Bay (NZ), Hungary* (EU), Kosovo (EU), Liechtenstein (EU), Malta* (EU), Manawatu-Wanganui (NZ), Missouri (US), Montana (US), Montenegro (EU), Newfoundland (CN), New Mexico (US), Northwest Territories (CN), Nunavut (CN), Northland (NZ), North Macedonia* (EU), Nova Scotia (CN), Otago (NZ), Rhode Island (US), San Marino (EU), South Carolina (US), South Dakota (US), Southland (NZ), Taranaki (NZ), Turkey* (EU), Ukraine (EU), Utah (US), Vermont (US), Waikato (NZ), Wellington (NZ), West Coast (CN), Wyoming (US) and Yukon (CN).

Legislative Unit	Implicit Total Ban for Advertising	Explicit Total Ban for Advertising	Partial Ban for Advertising	Regulations for Health Information	Source
Alberta (CN)	no	no	yes	no	Skin cancer prevention (artificial tanning) act Chapter S-7.9
Arizona (US)	no	no	no	yes	36-799.03. Senate Bill 1290
Auckland (NZ)	no	no	yes	no	Guidelines for Operators of Ultraviolet (UV) Tanning Lamps (basierend auf AS/NZS 2635:2008 Solaria for cosmetic purposes, 2.1; S. 1 Guidelines; Sun-beds code of practice How to provide a safe service—Promotion
Austria* (EU)	no	no	no	yes	Anlage 2 Solarienverordnung
Belgium* (EU)	no	no	no	yes	Arrêté royal relatif aux conditions d’exploitation des centres de bronzage Annexe II
California (US)	no	no	yes	yess	CA Bus and Prof Code § 22705 (2016)
Colorado (US)	no	no	no	yes	Artificial Tanning Consumer Warning
Delaware (US)	no	no	no	yes	Delaware Code relating to tanning facilities, Amendment to Title 16 (August 2009)
Denmark* (EU)	no	no	no	yes	Lov om solarier (LOV nr 718, 2014)
England (EU)	no	no	no	yes	Section 5 Sunbeds Regulation Act 2010
Finland* (EU)	no	no	no	yes	Radiation Act (859/2018)
Florida (US)	no	no	yes	yes	381.89 5 Regulation of tanning facilities
France* (EU)	no	no	yes	yes	Article 9 (Oct 2014); Article 12 and 13 decret 2013/1261; Article 14 decret 2013/1261
Georgia (US)	no	no	yes	yes	O.C.G.A. § 31-38-4
Germany* (EU)	no	no	no	yes	Addition 7 (for § 7 paragraph 1)
Hawai (US)	no	no	no	yes	Section 2f HB 1783 (Amendment to §321 Tanning facilities, 2014)
Iceland* (EU)	no	no	no	yes	Reglugerð um sólarlampa (810/2003)
Idaho (US)	no	no	yes	no	Legislation of the State Idaho, Housebill No. 268
Illionis (US)	no	no	yes	yes	Health facilities and regulation (210 ILCS 145/) Tanning Facility Permit Act
Indiana (US)	no	no	yes	yes	IC 25-8-15.4-13 Denial of risks
Iowa (US)	no	no	yes	yes	Training program for operators
Ireland* (EU)	no	no	yes	yes	9. Public Health (Sunbeds) Act 2014; 10. Public Health (Sunbeds) Act 2014; Annex VI, Guidance for Industry
Italy* (EU)	no	no	no	yes	2. Gazetta ufficiale
Kansas (US)	no	no	yes	yes	Article 19. Licensure of entities by state board of cosmetology
Kentucky (US)	no	no	no	yes	Section 217.924—Requirements for tanning facilities
Latvia* (EU)	no	no	yes	yes	Prasības kosmētiskā iedeguma pakalpojuma sniegšanai
Lithuania* (EU)	no	no	yes	yes	Del lietuvos Higienos Normos HN 71:2009 “Solariumai Sveikatos Saugos Reikalavimai” Patvirtinimo.
Louisiana (US)	no	no	yes	yes	§1319 Tanning Facility Regulations
Luxembourg* (EU)	no	no	yes	yes	Loi du 24 mai 2018 sur les conditions d’hygiène et de salubrité relatives à la pratique des techniques de tatouage par effraction cutanée, du perçage, du branding, cutting, ainsi que du bronzage UV
Maine (US)	no	no	yes	yes	Section 5 F, 2013 tanning rules
Manitoba (CN)	no	no	yes	no	The Public Health Act
Maryland (US)	no	no	no	yes	Section 20–106 Annotated Code of Maryland (Amendment in Senate Bill 299, Part D and E, 2019)
Massachusetts (US)	no	no	yes	yes	The Public Health Act
Michigan (US)	no	no	yes	yes	2017 US Use 10/02/2005 M10277 Registerd body bronze tanning centers LLC 12802 R
Minnesota (US)	no	no	no	yes	Minnesota Statutes 2020 325H.09 Penalty
Mississippi (US)	no	no	yes	yes	Mississippi Department of Health Part 14 General Sanitation, Title 15 Subpart 70—General Sanitation Regulations Chapter 6—Regulations for tanning facilities Rule 6.1.10
Moldova (EU)	no	no	no	yes	Norme sanitaro—igienice pentru saloane/centre de bronzare/solariu—Subsecțiunea Cerinţe pentru protecţia utilizatorilor a saloanelor/centrelor de bronzare, no. 31 and 32
Monaco (EU)	no	no	no	yes	Cent Cinquantieme année- N° 7.813 Le numéro JOURNAL DE MONACO Bulletin Officiel de la Principauté Journal Hebdomadaire paraissant le Vendredi
Nebraska (US)	no	no	no	yes	71-3907 Indoor Tanning Facility Act
Netherlands* (EU)	no	no	no	yes	NVWA Rapport Biological effects of ultraviolet radiation relevant to health with particular reference to sunbeds for cosmetic purpose
Nevada (US)	no	no	no	yes	NRS 597.762 (Tanning facilities) Owner or operator: Additional duties
New Brunswick (CN)	no	no	yes	no	Artificial Tanning Act, Chapter 2013, c.21
New Hampshire (US)	no	no	no	yes	New Hampshire Rev Stat § 313-A:30 (2015)
New Jersey (US)	no	no	yes	yes	8:28-3.13 Advertising and Promotion Tanning Rules NJ
New South Wales (AUS)	yes	no	no	no	Radiation Act
New York (US)	no	no	no	yes	Title 10 SubChapter I Environmental Health Part 72 Tanning Facilities Section 72–1.9
North Carolina (US)	no	no	yes	nyes	§ 104E-9.1 a 3, 10A NCAC 15.1411
North Dakota (US)	no	no	yes	yes	North Dekota Legislative Branch 23-39-03
Northern Ireland (EU)	no	no	yes	no	6. Sunbeds Act (Northern Ireland)
Northern Territory (AUS)	yes	no	no	no	Radiation Act
Norway* (EU	no	no	no	yes	Ny stråleverforskrift (2017)
Ohio (US)	no	no	no	yes	Ohio Revised Code Title 47 Occupations-Professions Chapter 4713 Cosmetologists Section 4713.08 Administrative rules Part 17d
Oklahoma (US)	no	no	yes	yes	State of Oklahoma 1st Session of the 43rd Legislature (1991) House Bill No. 1184
Ontario (CN)	no	no	yes	no	4. Skin Cancer Prevention Act (Tanning Beds), 2013
Oregon (US)	no	no	yes	yes	Public Health Division—Chapter 333 Division 119 Registration of Tanning Facilities Part 333-119-0050 and 333-119-0060
Pennsylvania (US)	no	no	no	yes	Section 5 Indoor Tanning Regulation Act
Poland* (EU)	no	yes	no	yes	Art. 4 Gesetz vom 15. September 2017
Portugal* (EU)	no	no	yes	yes	Art. 4 Verordnung 77-B
Prince Edward Island (CN)	no	no	yes	no	Prince Edward Island Public Health Act Tanning Facility Regulations
Quebec (CN)	no	no	yes	no	Act to prevent skin cancer caused by artifiacial tanning
Queensland (AUS)	yes	no	no	no	Radiation Act
Romania* (EU)	no	no	yes	yes	Ordin. No. 291/2016 din 10 martie 2016 privind aprobarea Normelor de igienă pentru saloanele/centrele de bronzar
Saskatchewan (CN)	no	no	yes	no	The Health Hazard Regulations Chapter P-37.1 Reg 10
Scotland (EU)	no	no	no	yes	Schedule 1, Regulation 3 Public Health (Scotland) Act 2008 (Sunbeds)
Serbia* (EU)	no	no	no	yes	1.4 Pravilnik o Posebnim Sanitarium Uslovima Koje Moraju Da Ispune Objekti U Kojima Se Pruzaju usluge Odzavanja Hig ijene, Nege I Ulepsavanja Lica I Tela (Sl. glasnik RS, br. 8-2019)
Slovakia* (EU)	no	no	no	yes	Zákon z 21. Júna 2007 o ochrane, podpore a rozvoji verejného udravia a o zmene a doplnení niektorých zákonov
Slovenia* (EU)	no	no	yes	yes	Pravilnik o minimalnih sanitarno zdravstvenih pogojih za opravljanje dejavnosti higienske nege in drugih podobnih dejavnosti
South Australia (AUS)	yes	no	no	no	Radiation Act
Spain* (EU)	no	no	yes	yes	Real Decreto 1002-2002, de 27 de septiembre, por el que se regula la venta y utilización de aparatos de bronceado mediante radiaciones ultravioletas
Sweden* (EU)	no	no	yes	yes	Strålsäkerhetsmyndighetens föreskrifter om solarier och artificiella solningsanläggningar
Swizerland* (EU)	no	no	no	yes	A 3.2 Vollzugshilfe zur Verwendung von Solarien
Tasmania (AUS)	yes	no	no	no	Radiation Act
Tennessee (US)	no	no	no	yes	Tennessee Code Ann. § 68-117-107,106 and 107
Texas (US)	no	no	yes	yes	145.007 Health and Safety Code Chapter 145. Tanning Facilities
Victoria (AUS)	yes	no	no	no	Radiation Act 2005
Virginia (US)	no	no	yes	yes	§ 59.1-310.5.H Code of Virginia Chapter 24.1
Wales (EU)	no	no	no	yes	Schedule 1 nach 7(1) The Sunbeds (Regulation) Act 2010 (Wales) Regulations 2011
Washington (US)	no	no	yes	yes	Section 6 Proposed Substitute House Bill 2652
Western Australia (AUS)	yes	no	no	no	Radiation Act
West Virgina (US)	no	no	no	yes	West Virginia Code Chapter 16 Public Health Article 45 Tanning Facilities Part 3 Operation standards
Wisconsin (US)	no	no	yes	yes	WI Stat § 463.25 (2021)

## References

[1] Arnold M., Singh D., Laversanne M., Vignat J., Vaccarella S., Meheus F., Cust A.E., de Vries E., Whiteman D.C., Bray F. (2022). Global Burden of Cutaneous Melanoma in 2020 and Projections to 2040. JAMA Dermatol..

[2] Leiter U., Keim U., Garbe C. (2020). Epidemiology of skin cancer: Update 2019. Adv. Exp. Med. Biol..

[3] Garbe C., Keim U., Gandini S., Amaral T., Katalinic A., Hollezcek B., Martus P., Flatz L., Leiter U., Whiteman D. (2021). Epidemiology of cutaneous melanoma and keratinocyte cancer in white populations 1943–2036. Eur. J. Cancer.

[4] Raimondi S., Suppa M., Gandini S. (2020). Melanoma Epidemiology and Sun Exposure. Acta Derm. Venereol..

[5] El Ghissassi F., Baan R., Straif K., Grosse Y., Secretan B., Bouvard V., Benbrahim-Tallaa L., Guha N., Freeman C., Galichet L. (2009). A review of human carcinogens—Part D: Radiation. Lancet Oncol..

[6] Boniol M., Autier P., Boyle P., Gandini S. (2012). Cutaneous melanoma attributable to sunbed use: Systematic review and meta-analysis. BMJ.

[7] Wehner M.R., Shive M.L., Chren M.M., Han J., Qureshi A.A., Linos E. (2012). Indoor tanning and non-melanoma skin cancer: Systematic review and meta-analysis. BMJ.

[8] An S., Kim K., Moon S., Ko K.P., Kim I., Lee J.E., Park S.K. (2021). Indoor Tanning and the Risk of Overall and Early-Onset Melanoma and Non-Melanoma Skin Cancer: Systematic Review and Meta-Analysis. Cancers.

[9] Gandini S., Sera F., Cattaruzza M.S., Pasquini P., Picconi O., Boyle P., Melchi C.F. (2005). Meta-analysis of risk factors for cutaneous melanoma: II. Sun exposure. Eur. J. Cancer.

[10] Whiteman D.C., Whiteman C.A., Green A.C. (2001). Childhood sun exposure as a risk factor for melanoma: A systematic review of epidemiologic studies. Cancer Causes Control..

[11] Gefeller O., Tarantino J., Lederer P., Uter W., Pfahlberg A.B. (2007). The relation between patterns of vacation sun exposure and the development of acquired melanocytic nevi in German children 6–7 years of age. Am. J. Epidemiol..

[12] Oliveria S.A., Saraiya M., Geller A.C., Heneghan M.K., Jorgensen C. (2006). Sun exposure and risk of melanoma. Arch. Dis. Child..

[13] Balk S.J. (2011). Ultraviolet radiation: A hazard to children and adolescents. Pediatrics.

[14] Fiessler C., Pfahlberg A.B., Keller A.K., Radespiel-Troger M., Uter W., Gefeller O. (2017). Association between month of birth and melanoma risk: Fact or fiction?. Int. J. Epidemiol..

[15] Gefeller O., Diehl K. (2022). Children and Ultraviolet Radiation. Children.

[16] Holman L.A.C. (2016). Indoor tanning promotions on social media in six US cities. Transl. Behav. Med..

[17] Naumovska L. (2017). Marketing Communication Strategies for Generation Y—Millennials. Bus. Manag. Strategy.

[18] Suppa M., Gandini S., Njimi H., Bulliard J.L., Correia O., Duarte A.F., Peris K., Stratigos A.J., Nagore E., Longo M.I. (2019). Prevalence and determinants of sunbed use in thirty European countries: Data from the Euromelanoma skin cancer prevention campaign. J. Eur. Acad. Dermatol. Venereol..

[19] Cokkinides V., Weinstock M., Lazovich D., Ward E., Thun M. (2009). Indoor tanning use among adolescents in the US, 1998 to 2004. Cancer.

[20] Guy G.P., Berkowitz Z., Jones S.E., Olsen E.O., Miyamoto J.N., Michael S.L., Saraiya M. (2014). State indoor tanning laws and adolescent indoor tanning. Am. J. Public. Health.

[21] Holman D.M., Jones S.E., Qin J., Richardson L.C. (2019). Prevalence of indoor tanning among US high school students from 2009 to 2017. J. Community Health.

[22] Reimann J., McWhirter J.E., Cimino A., Papadopoulos A., Dewey C. (2019). Impact of legislation on youth indoor tanning behaviour: A systematic review. Prev. Med..

[23] Garcez J. (2011). Battle for Brazil—The state vs. tanning. Skin. Cancer Found. J..

[24] Dessinioti C., Stratigos A.J. (2022). An Epidemiological Update on Indoor Tanning and the Risk of Skin Cancers. Curr. Oncol..

[25] Sinclair C.A., Makin J.K., Tang A., Brozek I., Rock V. (2014). The role of public health advocacy in achieving an outright ban on commercial tanning beds in Australia. Am. J. Public. Health.

[26] Rodriguez-Acevedo A.J., Green A.C., Sinclair C., Van Deventer E., Gordon L.G. (2020). Indoor tanning prevalence after the International Agency for Research on Cancer statement on carcinogenicity of artificial tanning devices: Systematic review and meta-analysis. Br. J. Dermatol..

[27] Pawlak M.T., Bui M., Amir M., Burkhardt D.L., Chen A.K., Dellavalle R.P. (2012). Legislation restricting access to indoor tanning throughout the world. Arch. Dermatol..

[28] Longo M.I., Bulliard J.L., Correia O., Maier H., Magnusson S.M., Konno P., Goad N., Duarte A.F., Olah J., Nilsen L.T.N. (2019). Sunbed use legislation in Europe: Assessment of current status. J. Eur. Acad. Dermatol. Venereol..

[29] Diehl K., Lindwedel K.S., Mathes S., Gorig T., Gefeller O. (2022). Tanning Bed Legislation for Minors: A Comprehensive International Comparison. Children.

[30] Jordan A., Wurzel R.K.W., Zito A. (2005). The rise of ‘new’policy instruments in comparative perspective: Has governance eclipsed government?. Political Stud..

[31] Glahn A., Kyriakos C.N., Loghin C.R., Nguyen D., Starchenko P., Jimenez-Ruiz C., Faure M., Ward B. (2018). Tobacco control achievements and priority areas in the WHO Europe Region: A review. Tob. Prev. Cessat..

[32] World Health Organization (2017). Sunbeds: National regulations on public information and safety. https://www.who.int/data/gho/data/themes/topics/indicator-groups/indicator-group-details/GHO/legislation-of-artificial-tanning-sunbeds---national-regulations-on-public-information-and-safety.

[33] Gosselin S., McWhirter J.E. (2019). Assessing the content and comprehensiveness of provincial and territorial indoor tanning legislation in Canada. Health Promot. Chronic Dis. Prev. Can..

[34] U.S. Food and Drug Administration Code of Federal Regulations Title 21—Food and Drugs Chapter I—Food and Drug Administration Department of Health and Human Services Subchapter J—Radiological Health (21CFR1040.20). https://www.accessdata.fda.gov/scripts/cdrh/cfdocs/cfcfr/cfrsearch.cfm?fr=1040.20.

[35] (2013). Household and Similar Electrical Appliances—Safety—Part 2–27: Particular Requirements for Appliances for Skin Exposure to Ultraviolet and Infrared Radiation.

[36] World Health Organization (2003). Artificial Tanning Sunbeds: Risks and Guidance.

[37] World Health Organization (2017). Artificial Tanning Devices: Public Health Interventions to Manage Sunbeds.

[38] Dellavalle R.P., Parker E.R., Cersonsky N., Hester E.J., Hemme B., Burkhardt D.L., Chen A.K., Schilling L.M. (2003). Youth access laws: In the dark at the tanning parlor?. Arch. Dermatol..

[39] Dellavalle R.P., Guild S. (2012). Additional restrictions of indoor UV tanning. Arch. Dermatol..

[40] Reimann J., McWhirter J.E., Papadopoulos A., Dewey C. (2018). A systematic review of compliance with indoor tanning legislation. BMC Public Health.

[41] Conte S., Aldien A.S., Jetté S., LeBeau J., Alli S., Netchiporouk E., Lagacé F., Lefrançois P., Iannattone L., Litvinov I.V. (2023). Skin Cancer Prevention across the G7, Australia and New Zealand: A Review of Legislation and Guidelines. Curr. Oncol..

[42] Orth B., Merkel C. (2019). Rauchen bei Jugendlichen und Jungen Erwachsenen in Deutschland. Ergebnisse des Alkoholsurveys 2018 und Trends.

[43] Geller A.C., Colditz G., Oliveria S., Emmons K., Jorgensen C., Aweh G.N., Frazier A.L. (2002). Use of sunscreen, sunburning rates, and tanning bed use among more than 10 000 US children and adolescents. Pediatrics.

[44] Warthan M.M., Uchida T., Wagner R.F. (2005). UV light tanning as a type of substance-related disorder. Arch. Dermatol..

[45] Levins P.C., Carr D.B., Fisher J.E., Momtaz K., Parrish J.A. (1983). Plasma beta-endorphin and beta-lipoprotein response to ultraviolet radiation. Lancet.

[46] Belon P.E. (1985). UVA exposure and pituitary secretion. Variations of human lipotropin concentrations (beta LPH) after UVA exposure. Photochem. Photobiol..

[47] Feldman S.R., Liguori A., Kucenic M., Rapp S.R., Fleischer A.B., Lang W., Kaur M. (2004). Ultraviolet exposure is a reinforcing stimulus in frequent indoor tanners. J. Am. Acad. Dermatol..

[48] Freeman B., Watts C., Astuti P.A.S. (2022). Global tobacco advertising, promotion and sponsorship regulation: What’s old, what’s new and where to next?. Tob. Control..

[49] Greenman J., Jones D.A. (2010). Comparison of advertising strategies between the indoor tanning and tobacco industries. J. Am. Acad. Dermatol..

[50] Sinclair C., Makin J.K. (2013). Implications of lessons learned from tobacco control for tanning bed reform. Prev. Chronic Dis..

[51] Holick M.F. (2008). Sunlight, UV-radiation, vitamin D and skin cancer: How much sunlight do we need?. Adv. Exp. Med. Biol..

[52] Webb A.R. (2006). Who, what, where and when-influences on cutaneous vitamin D synthesis. Prog. Biophys. Mol. Biol..

[53] Schulman J., Fisher D.E. (2009). Indoor ultraviolet tanning and skin cancer: Health risks and opportunities. Curr. Opin. Oncol..

[54] Jager N., Schope J., Wagenpfeil S., Bocionek P., Saternus R., Vogt T., Reichrath J. (2018). The Impact of UV-dose, Body Surface Area Exposed and Other Factors on Cutaneous Vitamin D Synthesis Measured as Serum 25(OH)D Concentration: Systematic Review and Meta-analysis. Anticancer. Res..

[55] Scientific Committee on Health Environmental and Emerging Risks (SCHEER) (2016). Opinion on Biological Effects of Ultraviolet Radiation Relevant to Health with Particular Reference to Sunbeds for Cosmetic Purposes.

[56] Lerche C.M., Philipsen P.A., Wulf H.C. (2017). UVR: Sun, lamps, pigmentation and vitamin D. Photochem. Photobiol. Sci..

[57] Herzinger T., Berneburg M., Ghoreschi K., Gollnick H., Hölzle E., Hönigsmann H., Lehmann P., Peters T., Röcken M., Scharffetter-Kochanek K. (2016). S1 guideline on UV phototherapy and photochemotherapy. J. Dtsch. Dermatol. Ges..

[58] Nilsen L.T., Aalerud T.N., Hannevik M., Veierod M.B. (2011). UVB and UVA irradiances from indoor tanning devices. Photochem. Photobiol. Sci..

[59] Erickson K.L., Fan C., Eley S.J., Vaccarello A., Xu J.R., Gendi S.W., Bordeaux J.S. (2023). UV safety recommendations and health claims by U.S. tanning salon operators in a nationally representative sample. J. Cosmet. Dermatol..

